# Adverse Childhood Experiences and Patient-Reported Outcome Measures in Critically Ill Children

**DOI:** 10.3389/fped.2022.923118

**Published:** 2022-07-13

**Authors:** Anna Rodenbough, Cydney Opolka, Tingyu Wang, Scott Gillespie, Megan Ververis, Anne M. Fitzpatrick, Jocelyn R. Grunwell

**Affiliations:** ^1^Department of Pediatrics, Emory University School of Medicine, Atlanta, GA, United States; ^2^Children’s Healthcare of Atlanta at Egleston, Atlanta, GA, United States

**Keywords:** adverse childhood experiences, pediatric, intensive care unit, patient-reported outcome measures, social determinants of health, anxiety, family relationships, national survey of children’s health

## Abstract

Adverse childhood experiences (ACEs) are linked to adverse health outcomes for adults and children in the United States. The prevalence of critically ill children who are exposed to ACEs is not known. Our objective was to compare the frequency of ACEs of critically ill children with that of the general pediatric population of Georgia and the United States using publicly available National Survey of Children’s Health (NSCH) data. The impact of ACEs on patient-reported outcome measures of emotional, social, and physical health in critically ill children is not known. We sought to determine whether a higher total number of ACEs was associated with poorer patient-reported measures of emotional, social, and physical health. We conducted a prospective cross-sectional study of children < 18 years of age who were admitted to a 36-bed free-standing, quaternary academic pediatric intensive care unit in Atlanta, Georgia from June 2020—December 2021. Parents of patients who were admitted to the pediatric intensive care unit completed a survey regarding their child’s ACEs, health care use patterns, and patient-reported outcome measures (PROMIS) of emotional, social, and physical health. Prevalence estimates of ACEs were compared with national and state data from the NSCH using Rao-Scott Chi-square tests. PROMIS measures reported within the PICU cohort were compared with population normed T-scores. The association of cumulative ACEs within the PICU cohort with patient-reported outcomes of emotional, social, and physical health were evaluated with a *t*-test. Among the 84 participants, 54% had ≥ 1 ACE, 29% had ≥ 2 ACEs, and 10% had ≥ 3 ACEs. Children with ≥ 2 ACEs had poorer anxiety and family relationship T-scores compared to those with ≤ 1 ACE. Given the high burden of ACEs in critically ill children, screening for ACEs may identify vulnerable children that would benefit from interventions and support to mitigate the negative effects of ACEs and toxic stress on emotional, social, and physical health.

## Introduction

Adverse childhood experiences (ACEs) are potentially traumatic events that occur in childhood. ACEs include witnessing or being a victim of violence in the home or community, being abused, neglected, or discriminated against, living in a household with substance abuse or mental health problems, or experiencing household instability due to parental separation, incarceration, or death (1, 2). Nearly half of all U.S. children have experienced at least one ACE; over 20% have experienced at least two ACEs (3). ACEs are linked to worse health outcomes in adulthood, including heart disease, cancer, asthma, traumatic brain injury, obesity, depression, substance abuse, and premature death (1, 2, 4–11). Adults with higher ACE exposures may attain lower educational and economic potential (8, 12). Data now links ACE exposure with poor health outcomes as early as childhood and adolescence (13, 14). While social determinants of health are associated with childhood admission to an intensive care unit and being more severely ill upon admission (15–18), there are no published studies that explicitly examine the link between ACEs and patient-reported outcome measures in critically ill children. Whether critically ill children have higher exposure to ACEs than the general pediatric population in Georgia and the U.S. is not known. Additionally, whether ACEs are associated with patient-reported outcome measures of emotional, social, and physical health in critically ill children is not known.

The primary objective of this study was to determine whether children admitted to an Atlanta, Georgia pediatric intensive care unit (PICU) had a higher exposure to ACEs compared to the general pediatric population in Georgia and the United States as reported by the National Survey of Children’s Health (NSCH) (19). We hypothesized that a higher proportion of children in the PICU would have more ACEs compared to Georgian or U.S. children. A secondary objective was to determine whether higher total ACE exposure was associated with poorer patient-reported outcomes of emotional, social, and physical health in the PICU cohort using the validated, population-normed PROMIS measures (20). We hypothesized that children with a higher total ACE exposure would have poorer patient-reported outcome measures of emotional, social, and physical health.

## Materials and Methods

### Study Design

This prospective, observational study was performed in the 36-bed academic medical/surgical Pediatric Intensive Care Unit (PICU) at Emory University/Children’s Healthcare of Atlanta at Egleston from June 2020 through December 2021.

### Ethics Statement

The study was approved by the Institutional Review Board at Children’s Healthcare of Atlanta (IRB00000643) and all methods were carried out in accordance with relevant guidelines and regulations in the Declaration of Helsinki. Informed consent was obtained from the parents of all subjects and assent was obtained from all patients 6–17 years old prior to data collection.

### Inclusion/Exclusion Criteria

All children aged 0–17 years old admitted to the PICU at our large, urban, academic, quaternary care center for critical illness or injury were eligible for this study and represent a convenience sample. Children were excluded from the study if they had any end-of-life care limits (i.e., Do Not Resuscitate or Withdrawal of Life-Sustaining Treatment orders) in place, if they were admitted to the PICU for routine post-operative monitoring, if they had never lived in a home setting (i.e., ex-premature or medically complex infants who had never been discharged from the hospital), if the PICU attending did not wish them to be enrolled, or if they had previously been enrolled in the study. All patients in the PICU were screened on days that trained study staff were available to enroll patients. No questionnaires were administered until written informed consent was given. Study materials were available in English and Spanish.

### National Survey of Children’s Health Sample

The 2018–2020 NSCH is directed by the Health Resources and Services Administration (HRSA) Maternal and Child Health Bureau (MCHB). The NSCH is a nationally representative survey of children ages 0–17 years of age living in non-institutional settings in the 50 states and the District of Columbia (21–23). The NSCH questionnaire is a valid measure of ACEs and a standardized assessment of health status and health care access (24). NSCH data is publicly available and deidentified; thus, it did not qualify as human subjects research and did not require institutional review board approval.

### Participant Characterization

Demographic information, comorbidities, and healthcare use were obtained by parent report and medical history from the electronic medical record (eMR) using the same question format as that used in the NSCH (19). Study data were collected and managed using REDCap (Research Electronic Data Capture) electronic data capture tools hosted at Children’s Healthcare of Atlanta. REDCap is a secure, web-based software platform designed to support data capture for research studies, providing (1) an intuitive interface for validated data capture; (2) audit trails for tracking data manipulation and export procedures; (3) automated export procedures for seamless data downloads to common statistical packages; and (4) procedures for data integration and interoperability with external sources (25, 26).

### Study Measures

The eight-item validated ACE questionnaire was used from the NSCH. The eight ACE questions were self-reported by medically and developmentally able participants who were 12—17 years old and proxy-reported by parents for children 0—17 years old.

We chose nine validated and population-normed Patient-Reported Outcomes Measurement Information System (PROMIS) short forms containing a total of 44 items across the domains of emotional, social, and physical health. We used PROMIS questionnaires to measure life satisfaction, meaning and purpose, positive affect, psychological stress experiences, anxiety, depressive symptoms, sleep disturbances, family relationships, and peer relationships. PROMIS measures were only measured from the PICU cohort and are not part of the NSCH; however, the PROMIS measures are normalized to population values. The population mean PROMIS T-score is 50 with a standard deviation of 10; a higher PROMIS T-score indicates that more of the concept being measured is present (20). The PROMIS measures were administered to medically and developmentally able patients ages 12–17 years old and/or their parents using the proxy forms for 6–17-year-olds. Validated PROMIS measures for children 0–5 years old across the selected domains were not available at the time of study initiation.

### Comparison of Self- and Proxy-Reported Outcome Measures

Self-reported and parent-proxy ACE and PROMIS measures within the PICU cohort were compared using a Pearson correlation coefficient. When both self- and proxy-reported PROMIS T-scores were available, self-reported measures were used in the analysis (27).

### Statistical Analysis

Statistical analyses were performed using SAS v9.4 (Cary, NC). Using complex survey design methodologies recommended by the NSCH (22, 28), ACE frequencies in the study cohort were compared to Georgia (GA) and U.S. population data, available from the NSCH, using appropriate GA- and U.S.-weighted percentages and compared to the PICU frequencies *via* Rao-Scott Chi-square tests. T-scores for the PICU sample were calculated for PROMIS measures following automated scoring in REDCap. Only participants with complete ACE or PROMIS data were analyzed. In addition to the complete case analysis, we performed a sensitivity analysis where we assumed that missing ACE data in the NSCH dataset meant that a particular ACE was not experienced. PROMIS T-scores were compared for children with ≤ 1 vs. ≥ 2 ACEs in the PICU sample using two-sample *t*-tests and Cohen’s *d* effect sizes, interpreted as small (0.2), moderate (0.5), and large (0.8). Missing values for demographic and medical history occurred in fewer than 10% of participants. All statistical tests were performed two-sided, and *p*-values < 0.05 were considered statistically significant.

## Results

### Study Population

**FIGURE 1:**
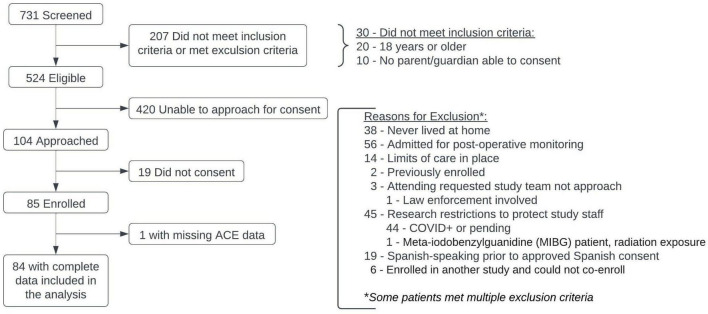
CONSORT diagram of PICU cohort.

A total of 731 children were screened for enrollment; 207 of those did not meet inclusion criteria or were excluded (Figure 1). There were 104 patients with parents/legal guardians who were available for consent in this convenience sample; a total of 85 patients were enrolled, with median time to enrollment of 2 days (IQR: 2–3.5) after PICU admission (Table 1). One patient less than 6 years of age did not have ACE data collected, leaving 84 patients available for analysis.

**TABLE 1:** Patient demographics and clinical characteristics.

Characteristic, *n* (%)*^a^*	PICU*^b,c^* *n* = 84	NSCH*^d^* GA *n* = 2,027	NSCH USA *n* = 102,740
**Age (y)**, ***n* (%)**			
0–5 6–11 12–17	41 (48.8) 21 (25.0) 22 (26.2)	536 (29.9) 621 (36.9) 870 (33.2)	28,891 (32.1) 31,493 (33.6) 42,356 (34.4)
**Sex, *n* (%)**			
Female Male	37 (44.1) 47 (55.9)	954 (49.0) 1,073 (50.9)	49,336 (48.9) 53,404 (51.1)
**Race, *n* (%)**			
White Black or African American American Indian or Alaska Native Asian Native Hawaiian/other Pacific Islander Other Multiple	28 (33.3) 53 (63.1) 0 (0.0) 0 (0.0) 0 (0.0) 0 (0.0) 3 (3.6)	1,340 (52.4) 390 (33.7) 13 (1.7) 129 (3.7) 3 (0.3) 22 (1.7) 130 (6.5)	79,360 (66.6) 7,348 (13.9) 945 (1.7) 5,418 (4.9) 582 (1.8) 834 (2.1) 8,253 (9.0)
**Ethnicity, *n* (%)**			
Hispanic or Latino Not Hispanic or Latino	4 (4.8) 80 (95.2)	210 (14.9) 1,817 (85.2)	12,946 (25.5) 89,794 (74.5)
**Insurance, *n* (%)**			
Public Private Private and public Not insured Missing	56 (68.3) 26 (31.7) 0 (0.0) 0 (0.0) 2	435 (30.9) 1,338 (53.7) 64 (4.7) 155 (10.8) 35	21,021 (30.1) 71,372 (58.4) 3,956 (4.6) 4,873 (6.9) 1,518
**Financial insecurity, *n* (%)**			
Never Rarely Somewhat often Very often Missing	49 (58.3) 19 (22.6) 13 (15.5) 3 (3.6) 0	1,133 (50.9) 610 (32.3) 197 (13.5) 41 (3.3) 46	57,919 (53.2) 30,915 (31.9) 9,695 (11.7) 2,216 (2.9) 1,995
**Comorbidities*^e^*, *n* (%)**			
0 1 2 +	21 (25.0) 35 (41.7) 28 (33.3)	1,157 (61.7) 459 (20.9) 411 (17.4)	61,292 (64.5) 21,429 (18.8) 20,019 (16.8)
PRISM score, median (IQR)	2 (0, 5)	n/a*^f^*	n/a
**Length of stay, median (IQR)**			
ICU days Hospital days	3 (2, 6) 5 (3, 9)	n/a	n/a
**Discharge disposition, *n* (%)**			
Inpatient rehabilitation Inpatient psychiatric facility Home	4 (4.8) 3 (3.6) 77 (91.7)	n/a	n/a
Time to Enrollment (days), median (IQR)	2 (2, 3.5)	n/a	n/a

*^a^Column percent does not account for missingness.*

*^b^Weight and stratum of 1 applied.*

*^c^PICU, pediatric intensive care unit.*

*^d^NSCH, National Survey of Children’s Health.*

*^e^Includes 28 comorbidities; missing comorbidities were coded as “no.”*

*^f^n/a, not applicable.*

Demographic and clinical history of the participants are shown in Table 1. Participants were compared with the demographic and clinical history of both Georgia and U.S. children surveyed in the NSCH. Notable differences in the PICU population studied compared with the NSCH GA and USA cohorts included a higher proportion of children who were in the 0 – 5 age range, male, Black or African American, non-Hispanic or Latino, and/or publicly insured (Table 1). Children in the PICU cohort had higher frequency of comorbid conditions such as asthma, hematologic or oncologic disorders, diabetes mellitus, and epilepsy or seizures as compared with the GA or USA NSCH cohorts (Table 1).

### Healthcare Use

A higher proportion of PICU children were never seen by a health care professional (13%) compared with children in GA (3.0%) and USA (4.1%) NSCH cohorts (Table 2). Of the children within the PICU cohort with no comorbidities, 42.9% of them had ≥ 2 ACEs compared to 57.1% of them who had < 2 ACEs (Supplementary Table 7). A higher proportion of PICU children were seen two or more times (56.1%) in the 12-months prior to PICU admission compared with children in the GA (37.6%) and United States (36.9%) NSCH cohorts (Table 2). Children within the PICU cohort with 2 or more comorbidities were more likely to be seen two or more times in the 12-months prior to PICU admission (77.8%) compared to those with one (51.4%) or no (35%) comorbidities (Supplementary Table 1). Of the children within the PICU cohort with two or more comorbidities, 28.6% experienced ≥ 2 ACEs compared to 71.4% who experienced < 2 ACEs (Supplementary Table 7). Children in the PICU were more likely to access medical care in a hospital emergency department or hospital outpatient center than children in the GA and U.S. NSCH cohorts (Table 2). Reasons for PICU admission are shown in Supplementary Table 2.

**TABLE 2:** Healthcare use for the children admitted to the pediatric intensive care unit compared with the general pediatric population in georgia or the united states from the national survey of children’s health.

Health care use, *n* (%)*^a^*	CHOA PICU*^b,c^* *n* = 84	NSCH*^d^* GA *n* = 2,027	NSCH USA *n* = 102,740
**Visit to health care professional in past 12 months**			
No–0 visit Yes–1 visit Yes–2 or more visits Missing (*n*)	11 (13.4) 25 (30.5) 46 (56.1) 2	58 (3.0) 1,066 (59.3) 581 (37.6) 322	3,726 (4.1) 55,098 (58.9) 27,349 (36.9) 16,567
**Has a place where usually receives sick care**			
Yes No Missing (*n*)	78 (93.9) 5 (6.0) 1	1,630 (76.7) 390 (23.3) 7	84,308 (76.9) 17,988 (23.1) 444
**Place where usually receives sick care**			
Doctor’s office Hospital ER Hospital outpatient Clinic or health center Retail store clinic School Some other place Missing (*n*)	53 (69.7) 16 (21.1) 4 (5.3) 3 (3.9) 0 (0.0) 0 (0.0) 0 (0.0) 8	1,476 (88.4) 23 (2.9) 3 (0.1) 71 (5.9) 25 (1.3) 6 (0.2) 17 (1.1) 406	72,626 (86.4) 739 (1.8) 452 (0.8) 8,317 (9.4) 774 (0.9) 271 (0.3) 437 (0.5) 19,124
**Has a place where receives preventive care**			
Yes No Missing (*n*)	78 (95.12) 4 (4.88) 2	1,867 (90.90) 144 (9.10) 16	95,850 (91.05) 6,318 (8.95) 572
**Receives sick and preventive care in same place**			
Yes No Missing (*n*)	73 (93.6) 5 (6.4) 6	1,773 (95.6) 85 (4.4) 169	91,066 (95.9) 4,282 (4.1) 7,392

*^a^Column percent does not account for missingness.*

*^b^Weight and stratum of 1 applied.*

*^c^PICU, pediatric intensive care unit.*

*^d^NSCH, National Survey of Children’s Health.*

### Adverse Childhood Experience Prevalence and Cumulative Burden in the Pediatric Intensive Care Unit vs. National Survey of Children’s Health Cohorts

**FIGURE 2:**
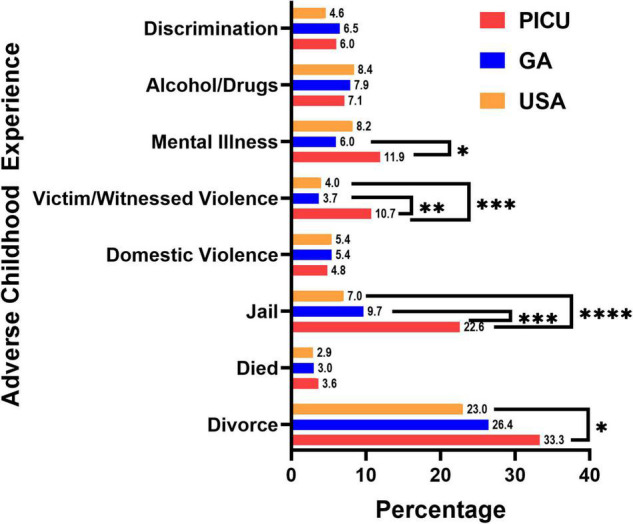
Adverse childhood experiences by type. Children admitted to the pediatric intensive care unit (PICU) had a higher percentage of divorced or separated parents, had a parent who served time in jail, witnessed or was a victim of neighborhood violence, and has lived with a person with mentally illness. Children admitted to the PICU (red bars, *n* = 84); weighted responses of children living in Georgia from the National Survey of Children’s Health (blue bars, *n* = 1,889); weighted responses of children throughout the United States (USA) from the National Survey of Children’s Health (orange bars, *n* = 97,173). Numbers at the top of the bars are the percentage of children experiencing that adverse childhood experience. Only complete cases were included in the analysis. Rao-Scott Chi-square tests were used to compare differences between groups. *p < 0.05, **p < 0.01, ***p < 0.001, ****p < 0.0001.

**FIGURE 3:**
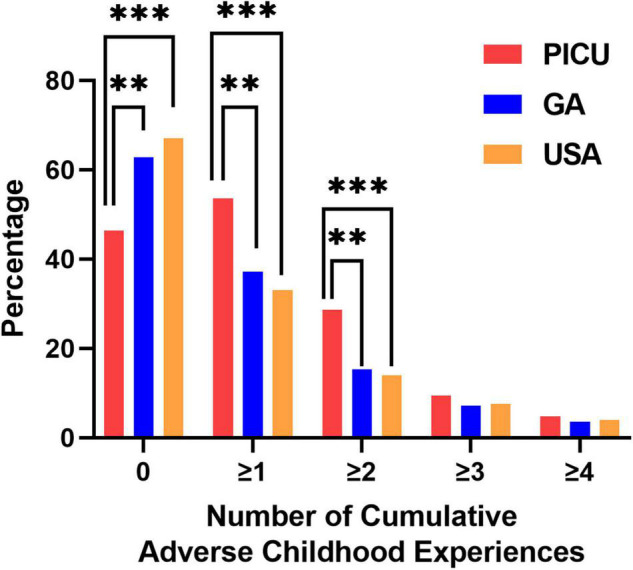
Number of cumulative adverse childhood experiences. Children admitted to the pediatric intensive care unit (PICU) had a higher percentage of experiencing ≥ 1 or ≥ 2 adverse childhood experiences compared to the general population of children living in Georgia or the United States. Children admitted to the PICU (red bars, *n* = 84); weighted responses of children living in Georgia from the National Survey of Children’s Health (blue bars, *n* = 1,889); weighted responses of children throughout the United States (USA) from the National Survey of Children’s Health (orange bars, *n* = 97,173). Only complete cases were included in the analysis. Rao-Scott Chi-square tests were used to compare differences between groups. **p < 0.01, ***p < 0.001.

We screened children admitted to the PICU for individual ACEs. As shown in Figure 2, children in the PICU were more likely to report experiencing parental/guardian separation or divorce, parental incarceration, witnessing or being a victim of neighborhood violence, or living with someone with a mental illness compared to children in the GA or U.S. NSCH populations (Figure 2). We next summed the number of ACEs experienced by each participant to determine whether children in the PICU had a higher cumulative ACE burden compared to the general pediatric population of GA and the U.S. surveyed in the NSCH. We found that a higher percentage of children in the PICU experienced ≥ 1 (vs. 0) and ≥ 2 (vs. ≤ 1) total ACEs compared with children in the GA or U.S. NSCH populations (Figure 3).

The age distribution of ACEs by age are shown in Supplementary Table 3. Older children in the PICU were more likely than younger children to report experiencing parental/guardian separation or divorce, parental incarceration, domestic violence, or witness or be a victim of neighborhood violence (Supplementary Table 3). Similarly, older children were more likely than younger children to have a higher total ACE burden (Supplementary Table 3).

Only complete cases in the NSCH were analyzed in our initial ACE analysis. To assess the degree of bias by analyzing only complete cases, we performed a sensitivity analysis where we assumed that missing ACE responses indicated no experience of an ACE. The results of this sensitivity analysis for the type and distribution of the total number of ACEs reported was the same as the complete case analysis (Supplementary Table 4).

### Patient Reported Outcome Measures

For children ages 6–17 years in the PICU cohort, we next evaluated nine domains of emotional, social, and physical health using the parent proxy- and/or self-reported PROMIS questionnaires (20). There were forty-three children ages 6–17 years with complete PROMIS measures available for analysis. There were 12 participants with both self- and proxy-reported PROMIS measures. We determined the correlation between the proxy- and self-reported PROMIS T-scores. In general, there was moderate linear association between the proxy- and self-reported PROMIS measures with Pearson correlation coefficients ranging from 0.55 to 0.76 (Supplementary Table 5). Measures with higher correlation (≥ 0.65) included meaning and purpose, positive affect, psychological stress, depressive symptoms, family relationships, and peer relationships (Supplementary Table 5). Measures with correlation < 0.65 included life satisfaction and anxiety (Supplementary Table 5). We report the summarized T-scores for the self- and proxy-reported PROMIS measures along with the combined overall cohort PROMIS T-score data, where self-reported T-scores were used if both self- and proxy-reported T-scores were available in Supplementary Table 6. The PROMIS T-scores for the overall PICU cohort were similar to the U.S. population normed T-score of 50 (*SD*: ± 10) with values ranging from 45.9 to 55.1; however, the standard deviation for each measure was somewhat variable and ranged from 8.0 to 11.2 (Supplementary Table 6).

**FIGURE 4:**
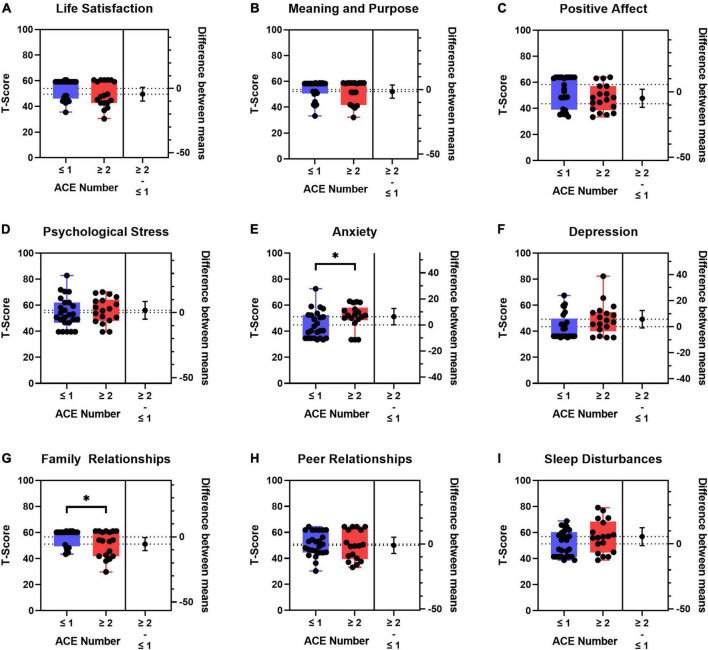
Boxplots of patient reported outcome measures (PROMIS) T-scores and difference between means by ≤ 1 (*n* = 25) vs. ≥ 2 (*n* = 18) adverse childhood experiences (ACEs). **(A)** Life Satisfaction, **(B)** meaning and purpose, **(C)** positive affect, **(D)** psychological stress, **(E)** anxiety, **(F)** depression, **(G)** family relationships, **(H)** peer relationships, and **(I)** sleep disturbances. *T*-tests were used to compare differences between groups. Self-reported PROMIS T-scores were used preferentially in children who also had proxy-reported PROMIS T-scores. If no self-reported PROMIS T-score was available, the proxy-reported T-score was used. Blue boxes denote ≤ 1 ACE; red boxes denote ≥ 2 ACEs. The population normed T-score is 50 with a standard deviation of 10. The higher the T-score means that there is more of the concept you are testing. Dotted horizontal lines are the means for the two groups. The lower and upper edges of the boxplots are the 25th and 75th percentiles, respectively. The whisker lines are the minimum and maximum values. The black circle to the right of each boxplot is the difference between means for the two groups with the 95% CI denoted by the whisker line. *p < 0.05.

We next assessed the PROMIS measures by total exposure to ACEs by splitting children into two groups that included children with ≤ 1 (*n* = 25) vs. ≥ 2 ACEs (*n* = 18) (Figure 4). The demographic and clinical characteristics by total ACE exposures are summarized in Supplementary Table 7. The subgroup of children with ≥ 2 ACEs was older and reported more financial insecurity, but there were no significant differences between the two subgroups with respect to sex, race, ethnicity, insurance type, comorbidities, illness severity, length of stay, or discharge disposition (Supplementary Table 7). Amongst children admitted to the PICU, those who had ≥ 2 ACEs had worse PROMIS scores than those with ≤ 1 ACE across multiple domains; however, significant differences were only seen in anxiety and family relationships (Figure 4). Mean differences and Cohen’s d criteria effect size were small (0.2) to moderate (0.5) for all of the PROMIS measures. The PROMIS measures with the largest Cohen’s d effect sizes when comparing ≤ 1 vs. ≥ 2 total ACEs were life satisfaction (0.45), positive affect (0.49), anxiety (0.64), depressive symptoms (0.57), family relationships (0.55), and sleep disturbances (0.56).

## Discussion

In this study, we compared the type and total number of ACEs in a cohort of critically ill children to publicly available National Survey of Children’s Health data from children living in Georgia and the United States. Consistent with our hypothesis, we found that a higher proportion of children in the PICU experienced ACEs compared to GA and U.S. children. We also used validated, population-normed patient-reported outcome measures with PROMIS questionnaires. We found wide variability in the PROMIS scores amongst children in the PICU; however, the PROMIS scores were similar to the mean population T-score. When we assessed PROMIS scores by total number of ACEs (≤ 1 vs. ≥ 2), we found that the anxiety and family relationship T-scores were worse for children with a higher ACE burden. Over half of the children in the PICU experienced at least one ACE, nearly 30% experienced two or more ACEs, and 10% experienced three or more ACEs. Children with a higher number of ACE exposures prior to their critical illness reported worse levels of anxiety and poorer family relationships than those with one or no ACEs.

Adverse childhood experiences are toxic stressors with long-term, negative consequences on health and life opportunities such as education and job potential (1, 2). The lasting, harmful risks associated with ACEs are well-known in adults with a higher cumulative ACE burden (1, 2). ACEs are associated with over forty different health, education, and employment opportunity (life) outcomes. Each cumulative ACE confers higher risk of worse health and life outcomes (2, 11).

The negative consequences of ACEs are not just limited to adults. Children who experience a higher total number of ACEs can have delayed brain development and weakened immune and stress-response systems (4, 29, 30). As a result, a child’s attention, decision-making, learning, and peer relationships may suffer (2). Our findings of worse PROMIS scores in critically ill children with 2 or more ACEs is consistent with published data showing worse mental health outcomes in adults who have more ACEs (1, 9, 10). The children in our PICU population are a vulnerable population at high risk for negative, lasting consequences of ACEs. We do not know whether a high baseline level of toxic stress affects susceptibility to critical illness, nor how critical illness interacts with ACEs to result in even greater levels of toxic stress and negative health outcomes. We do know that all children with critical illness are at high risk for significant morbidity and mortality even after initial recovery and hospital discharge (31, 32). We hope to further study the interactions between ACEs, critical illness, and outcomes in further studies; this includes the question of whether non-elective ICU admission itself should be considered an ACE.

As expected, the highest median cumulative ACE burden was seen in the oldest age group, as older children had more time to accrue ACEs. The PROMIS measures used in our analysis have been tested in children from 5 to 17 years of age with the measures showing factor invariance across age groups (33). Therefore, we did not adjust for age in our analysis of total ACE exposure with PROMIS measures. We did not include the PROMIS measures in the 0–5 age group due to the lack of availability of proxy-reported PROMIS measures in this age group at the time we began enrolling children into our study. Proxy-reported PROMIS questionnaires are now available for some measures, and future work should use these measures. The participants in our PICU cohort had similar overall levels of financial insecurity as reported for children in the GA and USA NSCH cohorts; however, there were large differences in health care use including greater use of a hospital emergency department as a primary place to seek both sick and well care. Our findings echo the results from a pediatric emergency department-based study of ACEs and healthcare use patterns from our Children’s Healthcare of Atlanta healthcare system (34). In this pediatric emergency department study of children who did not require acute stabilization, 28% of children reported experiencing 1 ACE and 18% experienced ≥ 2 ACEs. A greater proportion of children admitted to the PICU had even higher numbers of ACEs compared to the pediatric emergency department cohort. These findings underscore the importance of understanding the link between ACEs, severity of illness, and recovery from critical illness.

We believe it likely that ACE exposure of children in our PICU population is underestimated. First, we had a higher proportion of children 5 years and younger in our study cohort compared to the distribution of children in the NSCH. Older children have more time to accumulate ACEs. Second, we were not able to enroll children without a parent or guardian at the bedside, nor were we able to enroll children who were taken into state custody due to abuse, neglect, or other family instability. Third, some parents may have declined study participation out of the fear that disclosure of ACEs would result in legal ramifications and/or impact on their child’s medical care. There were also instances where parents acknowledged the presence of multiple ACEs to study staff but declined study participation citing the fear of reliving the trauma of the event(s) and experiencing post-traumatic stress. Lastly, some parental (proxy) ACE survey responses were discordant with additional ACEs reported to the medical team caring for the participant and recorded in the eMR.

The pediatric intensive care unit may be an effective and efficient place to implement ACE screening given the high proportion of critically ill children who reported experiencing at least one ACE. So, what can a pediatric intensivist do for the child and family once ACEs are disclosed? A growing body of research shows that there are effective interventions to prevent ACEs and mitigate their effects (2, 4, 30, 35). While the overall benefit of universal screening for ACEs in health care settings remains uncertain due to potential re-traumatization as well as limited understanding of best practices to address ACEs once they are identified, we suggest that screening is warranted if effective mitigation strategies can be employed. The Center for Disease Control and Prevention (CDC) has published a National Center for Injury Prevention and Control strategy aimed at identifying children with ACEs and preventing the accumulation of more ACEs using trauma-informed and evidence-based approaches (35). Pediatric intensivists can work to refer at-risk patients and families to programs and services including home visitation programs, enhanced primary care programs, mentoring programs, and trauma-focused cognitive behavioral therapy and multisystem therapy (2, 36). In addition, all pediatric providers can advocate for and support population-level interventions such as accessible early childhood education, violence prevention programs, youth-serving organizations, and multi-sector partnerships that work to identify and address ACEs (2, 35, 36). The pediatric ICU may serve as an important setting to trial whether ACE prevention and mitigation strategies can be implemented during hospitalization to improve the long-term outcomes and maximize the life opportunity potential of critically ill children. We believe it is especially important.

Patient reported outcome measures using PROMIS questionnaires have been validated for use in children with traumatic brain injury (37). The Common Data Elements project out of the National Institutes of Health National Institute of Neurological Disorders and Stroke encourages researchers to demonstrate the validity and utility of standardized instruments such as the PROMIS measures and to seek funding to support the validation and use of these metrics (38). Clinicians are adopting these measures into clinical practice, and PROMIS measures are integrated into some Epic eMR systems (39, 40). There is interest in expanding the use of validated and standardized patient-reported outcome measures and health-related quality of life questionnaires in pediatric critical care medicine (41–46). Further studies, such as ours, are needed to determine the most appropriate measures of overall health in the pediatric critically ill population (42, 45).

Our study has several limitations. First, children were enrolled at a single, quaternary academic medical center in Atlanta. Our hospital serves a predominantly minority, publicly insured population and our results may not be generalizable to other populations. Our study highlights the demographic differences in our PICU sample compared to the Georgia and national-weighted samples. We did not adjust for this in our descriptive analysis, and future work evaluating comparative ACE outcomes with regression adjustment or sample stratification may be used to account for demographic differences. ACEs are a sensitive topic that can induce stress when remembering past traumatic events. We were unable to enroll any children admitted due to immediate sequela of non-accidental trauma or children who are in the custody of the state. As a result, our cohort likely underestimates the frequency of ACEs in our PICU population due to selection bias. We were not able to approach a large proportion of eligible study participants for consent because the entire recruitment period occurred during the COVID-19 pandemic with associated limitations to non-COVID-19-related clinical research, restrictions on non-essential clinical research personnel working in-person, visitor restrictions, and the constraint of obtaining in-person consent from a parent/legal guardian. Analyses related to race/ethnicity within our PICU cohort are limited by collection of this data by hospital registration staff at the time of admission without subsequent verification by study staff at the time of enrollment. This may have led to simplified classification and/or misclassification of some subjects (47); however the participants in our study accurately reflect the demographics of children for whom we care in our PICU. It is known that a parent or guardian’s childhood exposure to ACEs can affect their children (12, 48, 49). We did not ask parents or guardians about their childhood ACE exposures to look for correlation with their child’s ACE and PROMIS measures. Exploring the interaction between a parent/guardian and child’s ACE exposure is a potential future direction of our work. Finally, Early Childhood Parent-Report PROMIS measures are now available for several of the domains including depressive symptoms, anxiety, positive affect, sleep disturbances, and family relationships. Future studies that can be expanded to incorporate PROMIS measures in children ages 0–5 years.

In summary, children admitted to the pediatric intensive care unit are exposed to higher-than-average ACEs. Hospitalization of a high-risk group of critically ill children provides a contact point whereby identification of ACEs *via* universal screening can be employed. Furthermore, strategies to mitigate the downstream effects of ACEs can be deployed to provide necessary family support for high-risk children. Better identification and understanding of ACEs could inform interventions aimed at reducing morbidity from toxic stress to narrow the disparity gaps in health and life outcomes in vulnerable children experiencing critical illness.

## Data Availability Statement

The raw data supporting the conclusions of this article will be made available by the authors, without undue reservation.

## Ethics Statement

The studies involving human participants were reviewed and approved by Children’s Healthcare of Atlanta Institutional Review Board. Written informed consent to participate in this study was provided by the participants’ legal guardian/next of kin.

## Author Contributions

AR, JG, and AF conceived and developed the study. AR and JG supervised the data acquisition and analyzed and interpreted the data, drafted and edited the manuscript. CO, MV, and AR enrolled participants and acquired the data. JG, TW, and SG performed the statistical analyses and edited the manuscript. All authors edited and approved the final version of this manuscript and agreed to be accountable for the content of the work.

## Conflict of Interest

The authors declare that the research was conducted in the absence of any commercial or financial relationships that could be construed as a potential conflict of interest.

## Publisher’s Note

All claims expressed in this article are solely those of the authors and do not necessarily represent those of their affiliated organizations, or those of the publisher, the editors and the reviewers. Any product that may be evaluated in this article, or claim that may be made by its manufacturer, is not guaranteed or endorsed by the publisher.

## Abbreviations

ACE: adverse childhood experiences
eMR: electronic medical record
IQR 25th to 75th: percentile interquartile range
NSCH: National Survey of Children’s Health
PICU: pediatric intensive care unit
PROMIS: Patient-Reported Outcomes Measurement Information System.
