# Supplementary material for: Adverse Childhood Experiences and Patient-Reported Outcome Measures in Critically Ill Children
Source: Front Pediatr. 2022 Jul 13;10:923118. doi: 10.3389/fped.2022.923118 (PMC9326064; doi:10.3389/fped.2022.923118)
Supplement: Supplementary file 1 [file Data_Sheet_1.pdf]

Adverse Childhood Experiences and Patient-Reported Outcome Measures in Critically Ill Children

Anna Rodenbough, Cydney Opolka, Tingyu Wang, Scott Gillespie, Megan Ververis, Anne M. Fitzpatrick, and Jocelyn R. Grunwell

Supplementary Digital Material

Table E1. Health Care Use within Pediatric Intensive Care Unit (PICU) Cohort by Number of Comorbidities

Table E2. Pediatric Intensive Care Unit (PICU) Admission Diagnosis

Table E3. Adverse Childhood Experience (ACE) Exposures in the Pediatric Intensive Care Cohort by Age

Table E4. Adverse Childhood Experience by Type and Cumulative Burden

Table E5. Correlation Between Parent (Proxy)-Reported and Self-Reported Patient Reported Outcome Measure T-Scores

Table E6. Patient Reported Outcome Measure (PROMIS) T-Scores for Children by Self- or Proxy-Report

Table E7. Patient Demographics and Clinical Characteristics for Children with ≤ 1 versus ≥ 2 Adverse Childhood Experiences

Table E1. Health Care Use within Pediatric Intensive Care Unit (PICU) Cohort by Comorbidities

Health Care Use, n (%)[a]	0 comorbidities[b] N=21	1 comorbidity N=35	≥ 2 comorbidities N=28	*p* value
Visit to health care professional in past 12 months				
No – 0 visit	7 (35%)	3 (8.6%)	1 (3.7%)	
Yes – 1 visit	6 (30%)	14 (40%)	5 (18.5%)	0.006
Yes – 2 or more visits	7 (35%)	18 (51.4%)	21 (77.8%)	
Missing (n)	1	0	1	

[a]Column percent does not account for missingness
[b]Includes 28 comorbidities; missing comorbidities were coded as “no”

Table E2. Pediatric Intensive Care Unit (PICU) Admission Diagnosis

Admission diagnosis, *n* (%)	PICU *n* = 84
Cardiology	1 (1.2)
Electrolyte Abnormality	1 (1.2)
Endocrinology	12 (14.5
Diabetes	10 (12.1)
Gastroenterology/Anemia	1 (1.2)
Hematology	16 (19.3)
Oncology	11(13.3)
Ingestion	8 (9.6)
Accidental	2 (2.4)
Intentional	5 (6.0)
Nephrology	1 (1.2)
Neurology	6 (7.2)
Seizure	4 (4.8)
Respiratory	25 (30.1)
Acute respiratory failure	7 (8.4)
Asthma	12 (14.5)
Bronchiolitis	4 (4.8)
Pneumonia	2 (2.4)
Near Drowning	1 (1.2)
Traumatic Injury	11 (13.3)
Gunshot wound	5 (6.0)
Brain	2 (2.4)
Envenomation	1 (1.2)
Missing (*n*)	1 (1.2)

Table E3. Adverse Childhood Experience (ACE) Exposures in the Pediatric Intensive Care Cohort by Age

		Years			
**Adverse childhood experience, *n* (%)**[a]	**Overall *n* = 84**	**0-5 *n* = 41**	**6-11 *n* = 21**	**12-17 *n* = 22**	***p*-value**[b]
Parent or guardian divorced or separated	28 (33.3)	8 (19.5)	8 (38.1)	12 (54.6)	0.017
Parent or guardian died	3 (3.6)	1 (2.4)	2 (9.5)	0 (0.0)	0.220
Parent or guardian served time in jail	19 (22.6)	5 (12.2)	3 (14.3)	11 (50.0)	0.003
Saw or heard parents or adults slap, hit, kick, punch one another in the home	4 (4.8)	0 (0.0)	1 (4.8)	3 (13.6)	0.039
Was a victim of violence or witnessed violence in his or her neighborhood	9 (10.7)	1 (2.4)	4 (19.1)	4 (18.2)	0.029
Lived with anyone who was mentally ill, suicidal, or severely depressed	10 (11.9)	2 (4.9)	3 (14.3)	5 (22.7)	0.099
Lived with anyone who had a problem with alcohol or drugs	6 (7.1)	1 (2.4)	1 (4.8)	4 (18.2)	0.086
Treated or judged unfairly because of his or her race or ethnic group	5 (5.9)	2 (4.9)	1 (4.8)	2 (9.1)	0.841
Total ACE burden (median, 25th ,75th)	1 (0, 2)	0 (0, 1)	1 (0, 1)	2 (1, 3)	<.0001
Total ACE burden (mean, SD)	1 (1.3)	0.5 (0.7)	1.1 (1.5)	1.9 (1.3)	<.0001

[a]Column percent

[b]Fisher's exact test for single experience question, Kruskal-Wallis (equal variance) for median ACE burden, One-way ANOVA (equal variance) for mean ACE burden

Table E4. Adverse Childhood Experience by Type and Cumulative Burden

Adverse childhood experience, *n* (%)	PICU *n* = 84	NSCH GA[a] *n* = 2027	*p*-value	NSCH USA[a] *n* = 102740	*p*-value
Parent or guardian divorced or separated	28 (33.3)	436 (25.3)	0.105	22767 (22.4)	0.016
Parent or guardian died	3 (3.6)	63 (2.9)	0.711	2971 (2.9)	0.691
Parent or guardian served time in jail	19 (22.6)	135 (9.4)	0.0001	6316 (6.9)	<.0001
Saw or heard parents or adults slap, hit, kick, punch one another in the home	4 (4.8)	83 (5.1)	0.879	5058 (5.3)	0.841
Was a victim of violence or witnessed violence in his or her neighborhood	9 (10.7)	56 (3.7)	0.003	3685 (3.9)	0.002
Lived with anyone who was mentally ill, suicidal, or severely depressed	10 (11.9)	137 (5.9)	0.032	9134 (7.8)	0.162
Lived with anyone who had a problem with alcohol or drugs	6 (7.1)	152 (7.5)	0.915	9582 (8.1)	0.754
Treated or judged unfairly because of his or her race or ethnic group	5 (5.9)	100 (6.4)	0.869	3806 (4.5)	0.505
Cumulative ACE burden					
0	39 (46.4)	1370 (62.8)	0.003	68994 (67.0)	<.0001
>= 1	45 (53.6)	657 (37.2)		33746 (32.9)	
<= 1	60 (71.4)	1755 (84.7)	0.002	88083 (85.9)	0.0001
>= 2	24 (28.6)	272 (15.3)		14657 (14.0)	
<= 2	76 (90.5)	1902 (92.8)	0.437	95018 (92.4)	0.505
>= 3	8 (9.5)	125 (7.2)		7722 (7.6)	
<= 3	80 (95.2)	1966 (96.4)	0.597	98711 (96.0)	0.717
>= 4	4 (4.8)	61 (3.6)		4029 (3.9)	
<= 4	82 (97.6)	1996 (98.2)	0.723	100698 (98.1)	0.756
>= 5	2 (2.4)	31 (1.8)		2042 (1.9)	
<= 5	83 (98.8)	2012 (98.9)	0.882	101869 (99.1)	0.753
>= 6	1 (1.19)	3 (1.0)		871 (0.9)	
<= 6	84 (100.0)	2026 (99.9)	-	102522 (99.8)	-
>= 7	0 (0.0)	1 (0.1)		218 (0.2)	
<= 7	84 (100.0)	2027 (100.0)	-	102706 (99.9)	-
>= 8	0 (0.0)	0 (0.0)		34 (0.04)	

[a]Missing values are assumed to be “no”

Table E5. Correlation Between Parent (Proxy)-Reported and Self-Reported Patient Reported Outcome Measure T-Scores

Parent	Self	N	Correlation (95% CI)[a,b]
Life Satisfaction	Life Satisfaction	12	0.55 (-0.04, 0.85)
Meaning and Purpose	Meaning and Purpose	12	0.74 (0.28, 0.92)
Positive Affect	Positive Affect	12	0.75 (0.31, 0.92)
Psychological Stress Experiences	Psychological Stress Experiences	12	0.75 (0.30, 0.92)
Anxiety	Anxiety	12	0.56 (-0.01, 0.86)
Depressive Symptoms	Depressive Symptoms	12	0.69 (0.19, 0.91)
Family Relationships	Family Relationships	12	0.76 (0.34, 0.93)
Peer Relationships	Peer Relationships	12	0.65 (0.13, 0.89)
Sleep Disturbances	Sleep Disturbances	12	0.80 (0.41, 0.94)

[a]CI = confidence interval
[b]Pearson correlation coefficient

Table E6. Patient Reported Outcome Measure (PROMIS) T-Scores for Children by Self- or Proxy-Report

PROMIS measure, mean T-score (SD[a])	Overall cohort
**Self- or Proxy-report[b]**	**n = 43[c]**
Life satisfaction	52.1 (8.8)
Meaning and purpose	52.8 (8.0)
Positive affect	50.6 (11.2)
Psychological stress experiences	55.1 (10.8)
Anxiety	47.5 (10.4)
Depressive symptoms	45.9 (11.0)
Family relationships	54.3 (8.4)
Peer relationships	50.6 (9.9)
Sleep disturbances	53.6 (11.2)
**Self-report only**	**n = 17**
Life satisfaction	50.7 (9.6)
Meaning and purpose	53.8 (8.3)
Positive affect	48.9 (10.7)
Psychological stress experiences	56.7 (9.5)
Anxiety	47.5 (10.7)
Depressive symptoms	49.1 (13.7)
Family relationships	53.3 (9.7)
Peer relationships	50.7 (10.4)
Sleep disturbances	55.2 (12.9)
**Parent-report only**	**n = 38**
Life satisfaction	52.5 (8.8)
Meaning and purpose	51.1 (9.2)
Positive affect	51.5 (12.3)
Psychological stress experiences	55.1 (11.5)
Anxiety	48.0 (11.6)
Depressive symptoms	44.6 (10.4)
Family relationships	54.4 (8.7)
Peer relationships	49.8 (9.6)
Sleep disturbances	54.0 (10.7)

[a]Self-reported PROMIS T-scores were used if both self- and proxy-reported T-scores were available
[b]SD = standard deviation
[c]Only complete cases are included

Table E7. Patient Demographics and Clinical Characteristics for Children with ≤ 1 versus ≥ 2 Adverse Childhood Experiences (ACEs)

Characteristic, n (%)[a]	ACE < 2 N=60	ACE ≥ 2 N=24	*p* value
Age (y), *n* (%)			
0-5	36 (60%)	5 (20.8%)	
6-11	16 (26.7%)	5 (20.8%)	**<0.001**
12-17	8 (13.3%)	14 (58.3%)	
Sex, *n* (%)			
Female	25 (41.7%)	12 (50%)	0.487
Male	35 (58.3%)	12 (50%)	
Race, *n* (%)			
White	18 (30%)	10 (41.7%)	
Black or African American	39 (65%)	14 (58.3%)	
American Indian or Alaska Native	0 (0%)	0 (0%)	
Asian	0 (0%)	0 (0%)	0.442
Native Hawaiian/Other Pacific Islander	0 (0%)	0 (0%)	
Other	0 (0%)	0 (0%)	
Multiple	3 (5%)	0 (0%)	
Ethnicity, *n* (%)			
Hispanic or Latino	4 (6.7%)	0 (0%)	0.321
Not Hispanic or Latino	56 (93.3%)	24 (100%)	
Insurance, *n* (%)			
Public	38 (65.5%)	18 (75%)	
Private	20 (34.5%)	6 (25%)	
Private and public	0 (0%)	0 (0%)	0.401
Not insured	0 (0%)	0 (0%)	
Missing	2	0 (0%)	
Financial insecurity, *n* (%)			
Never	39 (65%)	10 (41.7%)	
Rarely	13 (21.7%)	6 (25%)	
Somewhat often	8 (13.3%)	5 (20.8%)	**0.027**
Very often	0 (0%)	3 (12.5%)	
Missing	0	0	
Comorbidities[b], *n* (%)			
0	12 (20%)	9 (37.5%)	
1	28 (46.7%)	7 (29.2%)	0.186
2+	20 (33.3%)	8 (33.3%)	
PRISM score, *median (IQR)*	3.0 (0.0, 5.0)	0.0 (0.0, 4.0)	0.086
Length of stay, *median (IQR)*			
ICU days	3.5 (2, 6.5)	3 (2, 4.5)	0.307
Hospital days	5 (3, 9)	5 (2, 12.5)	0.687
Discharge disposition, *n* (%)			
Inpatient rehabilitation	2 (3.3%)	2 (8.3%)	0.574
Inpatient psychiatric facility	1 (1.7%)	2 (8.3%)	0.195
Home	57 (95%)	20 (83.3%)	0.099

[a]Column percent does not account for missingness
[b]Includes 28 comorbidities; missing comorbidities were coded as “no”
